# Supplementary material for: Neural correlates of ingroup bias for prosociality in rats
Source: eLife. 2021 Jul 13;10:e65582. doi: 10.7554/eLife.65582 (PMC8277352; doi:10.7554/eLife.65582)
Supplement: Supplementary file 1. [file elife-65582-supp1.docx]

Supplementary File 1. List of brain regions

| Region | Description |
| --- | --- |
| Pir1 | Primary Piriform cortex |
| Pir2 | Secondary Piriform cortex |
| Aud | Auditory cortex |
| S2 | Secondary somatosensory cortex |
| M1 | Primary Motor cortex |
| M2 | Secondary Motor cortex |
| TeA | Temporal association cortex |
| DEn | Dorsal endopiriform nucleus |
| ACC | Anterior cingulate cortex |
| PrL | Prelimbic cortex |
| LO | Lateral orbitofrontal cortex |
| VO | Ventral orbitofrontal cortex |
| MO | Medial orbitofrontal cortex |
| AID | Dorsal agranular insula |
| AIV | Ventral agranular insula |
| DCl | Dorsal claustrum |
| VCl | Ventral claustrum |
| BLA | Basolateral amygdala |
| BMA | Basomedial amygdala |
| LaAmy | Lateral amygdala |
| CeC | Central amygdaloid nucleus, capsular |
| CeL | Central amygdaloid nucleus, lateral |
| DG | Dentate gyrus |
| CA1 | CA1 of hippocampus |
| CA2 | CA2 of hippocampus |
| CA3 | CA3 of hippocampus |
| LS | Lateral septum |
| VDB | Nucleus vertical limb diagonal band |
| Cpu | Caudate putamen |
| ICj | Islands of Calleja |
| NacC | Nucleus accumbens core |
| NacSh | Nucleus accumbens shell |
| DMD | Dorsomedial hypothalamic nucleus, diffuse |
| IMD | Intermediodorsal thalamic nucleus |
| VMH | Ventromedial hypothalamic nucleus |
| ArcM | Medial arcuate hypothalamus |
| MEE | Medial eminence |
| PV | Paraventricular thalamic nucleus |
| Re | Reuniens thalamic nucleus |
| CM | Central median thalamic nucleus |
| Lhab | Lateral habenula |
| Mhab | Medial habenula |
| LPAG | Periaqueductal gray |
| SNR | Substantia nigra, reticular |
| VTA | Ventral tegmental area |
