# Supplementary material for: Neural correlates of ingroup bias for prosociality in rats
Source: eLife. 2021 Jul 13;10:e65582. doi: 10.7554/eLife.65582 (PMC8277352; doi:10.7554/eLife.65582)

Supplementary File 2. Means and confidence intervals for brain-wide c-Fos numbers across conditions


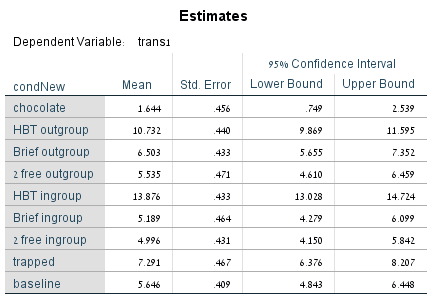

Supplement: Supplementary file 2. [file elife-65582-supp2.docx]
