# Supplementary figures and images for: Neural correlates of ingroup bias for prosociality in rats
Source: eLife. 2021 Jul 13;10:e65582. doi: 10.7554/eLife.65582 (PMC8277352; doi:10.7554/eLife.65582)

Supplementary File 3. Means and confidence intervals for c-Fos numbers per brain region
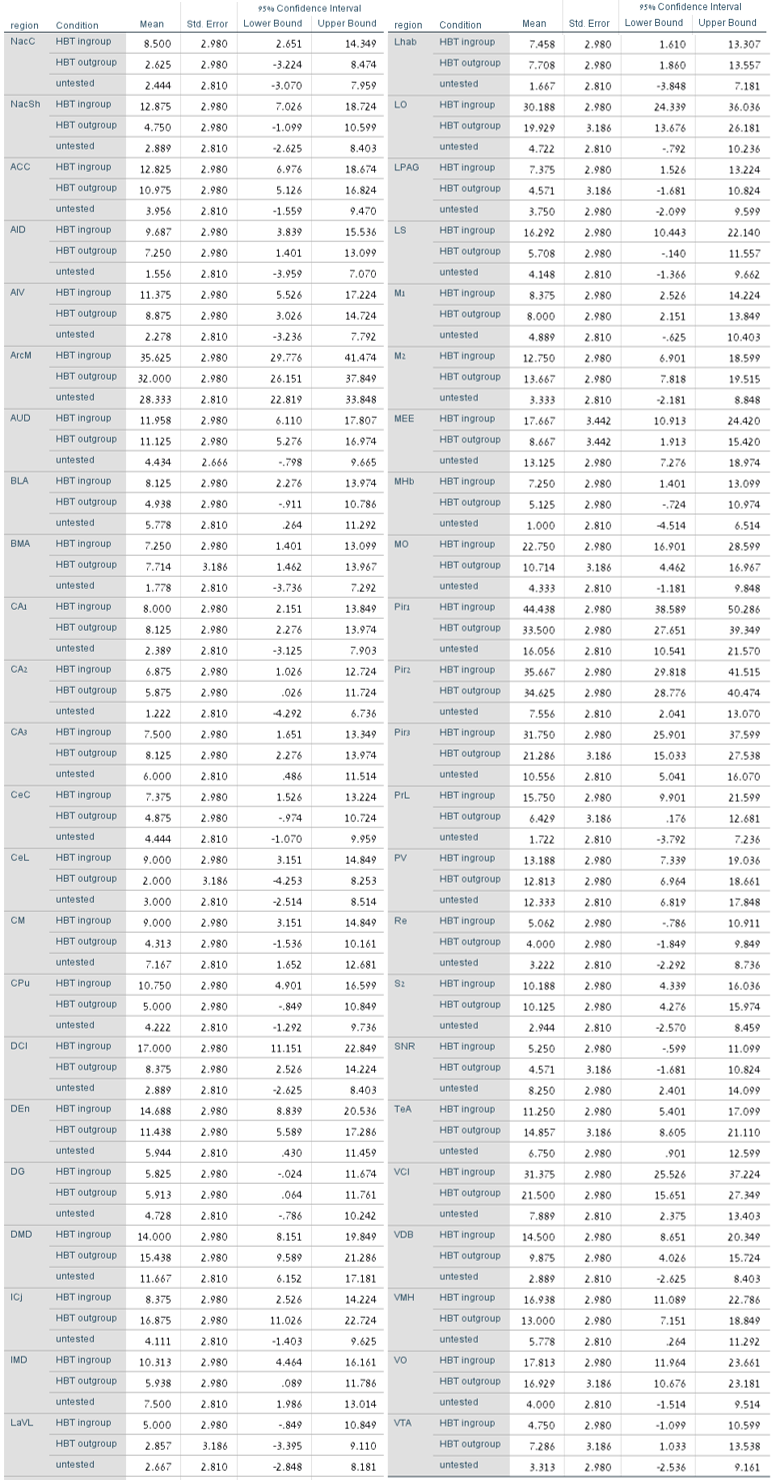

Supplement: Supplementary file 3. [file elife-65582-supp3.docx]
