# Supplementary material for: Neural correlates of ingroup bias for prosociality in rats
Source: eLife. 2021 Jul 13;10:e65582. doi: 10.7554/eLife.65582 (PMC8277352; doi:10.7554/eLife.65582)
Supplement: Supplementary file 4. — Combinations of clustering algorithms, parameters, and subsets of data used in Figure 3D. ‘Cluster group’ was used to obtain the cluster assignments for each brain region. ‘Reference group’ was the control group in the multinomial logistic regression. The ‘weighted’ column indicates whether weights (correlations) between brain regions were taken into account while performing clustering. ‘All’: all groups; ‘rest’: all conditions except ingroup and outgroup; ‘baseline’: untested baseline condition. The thresholds of percentile rank, p-value, and absolute r value in the parameter column were applied to the covariance matrix of the c-Fos data in the cluster group before feeding it into the selected clustering algorithm. [file elife-65582-supp4.docx]

Supplementary File 4. Set of parameters used in a series of multinomial regression tests
